# Generation and functional analysis of melanoma antigen‐specific CD8+ T cells derived from S/MAR vector‐transfected human induced pluripotent stem cells

**DOI:** 10.1002/ijc.35524

**Published:** 2025-06-12

**Authors:** Juliane Poelchen, Sandra Pardo, Daniel Novak, Qian Sun, Tamara Steinfass, Marlene Vierthaler, Özge Cicek Sener, Karol Granados Blanco, Yiman Wang, Jan Peter Nicolay, Pierre Guermonprez, Richard Harbottle, Viktor Umansky, Jochen Utikal

**Affiliations:** ^1^ Skin Cancer Unit German Cancer Research Center (DKFZ) Heidelberg Germany; ^2^ Department of Dermatology, Venereology and Allergology University Medical Center Mannheim, Ruprecht Karl University of Heidelberg Mannheim Germany; ^3^ DKFZ‐Hector Cancer Institute The University Medical Center Mannheim Mannheim Germany; ^4^ Faculty of Biosciences Ruprecht Karl University of Heidelberg Heidelberg Germany; ^5^ Department of Microbiology New York University Grossman School of Medicine New York USA; ^6^ Department of Biochemistry School of Medicine, University of Costa Rica (UCR) San Jose Costa Rica; ^7^ Section of Clinical and Experimental Dermatology Medical Faculty Mannheim, Heidelberg University Mannheim Germany; ^8^ Centre for Inflammation Research Université de Paris Paris France; ^9^ DNA Vector Laboratory German Cancer Research Center Heidelberg Germany; ^10^ Mannheim Institute for Innate Immunoscience (MI3) Medical Faculty Mannheim, Ruprecht Karl University of Heidelberg Mannheim Germany

**Keywords:** adoptive cell therapy, hiPSCs. S/MAR vector, melanoma, T cells

## Abstract

Melanoma accounts for the majority of all skin cancer‐related deaths with rising incidence rates. Adoptive cell therapies (ACT) with tumor antigen‐specific CD8+ T cells derived from human‐induced pluripotent stem cells (hiPSCs) might offer a promising treatment strategy for advanced malignant melanoma patients. In this study, we investigated two strategies for the generation of CD8+ T cells from hiPSCs expressing a T cell receptor (TCR) specific for the melanoma‐associated antigen recognized by T cells (MART‐1) or a chimeric antigen receptor (CAR) specific for the melanoma‐associated chondroitin sulfate proteoglycan (MCSP), respectively. While the long‐term co‐culture of bioengineered OP9 stromal cells with CD34+ hematopoietic stem/progenitor cells (HSPCs) facilitated the generation of CD4 + CD8+ double‐positive (DP) T cells, we encountered difficulties in obtaining high percentages of CD8+ single‐positive (SP) T cells using this method. However, the replacement of the OP9 cells with a T cell differentiation kit enabled the generation of CD8+ SP T cells after 47 days. Despite a low expression of the T cell marker CD3 on the surface of the generated CD8+ SP T cells, we detected intracellular IFN‐γ and surface CD107a expression upon stimulation. Moreover, the generated CD8+ SP T cells exhibited cytotoxic effects when co‐cultured with melanoma cell lines. The use of scaffold/matrix attachment region (S/MAR) DNA vectors ensured persistent expression of the TCR or the CAR during differentiation of T cells. Hence, these findings demonstrate the potential as well as the challenges associated with using S/MAR DNA vector‐transfected hiPSCs for the generation of melanoma antigen‐specific CD8+ T cells.

## Graphical abstract

What's new?

Adoptive cell therapy (ACT) with tumor antigen‐specific CD8+ T cells has shown success in clinical trials treating patients with advanced or metastatic melanoma. Here, the authors established protocols for generating CD8+ T cells from human induced pluripotent stem cells (hiPSCs) expressing a melanoma‐specific T cell receptor (TCR) or chimeric antigen receptor (CAR) construct. They transfected the cells with scaffold/matrix attachment region (S/MAR) DNA vectors, which helped ensure persistent expression of the TCR or CAR during differentiation. They showed that in vitro generation of CD8+ single‐positive T cells has promise but highlighted several challenges that remain.

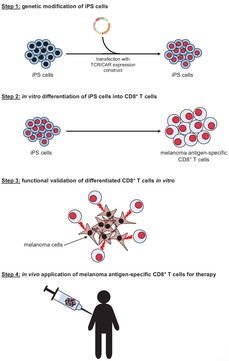

**Abbreviations**
ACT: adoptive cell therapy
ATOs: artificial thymic organoids
B2M: β2‐microglobulin
bFGF: basic fibroblast growth factor
CAR: chimeric antigen receptor
CGAs: cancer germline antigens
DN: double‐negative
DP: double‐positive
dpt: days post transfection
EBs: embryoid bodies
ECM: extracellular matrix
FBS: fetal bovine serum
FMO: fluorescence minus one
GATA3: GATA‐binding protein 3
GOI: gene of interest
gp100: glycoprotein 100
hiPSCs: human induced pluripotent stem cells
HSPCs: hematopoietic stem/progenitor cells
LEF1: lymphoid enhancer‐binding factor 1
MART‐1: melanoma‐associated antigen recognized by T cells
MCSP: melanoma‐associated chondroitin sulfate proteoglycan
NEAA: non‐essential amino acids
PMA: phorbol myristate acetate
ROCK: rho‐associated protein kinase
scFv: single chain variable fragment
SD: standard deviation
S/MAR: scaffold/matrix attachment region
SP: single‐positive
TCF1: T cell factor‐1
TCR: T cell receptor
TILs: tumor‐infiltrating lymphocytes

## 1 INTRODUCTION

Due to the high capacity to metastasize and to develop resistances against current therapies, melanoma counts as the most aggressive and deadliest skin cancer with a rising incidence rate of newly diagnosed cases every year.^1^, ^2^, ^3^ Adoptive cell therapy (ACT) using tumor antigen‐specific CD8+ T cells is a promising approach to treat patients with advanced or metastasizing melanoma showing success in clinical trials.^4^ However, challenges such as sudden disease relapse persist, mainly due to T cell dysfunction characterized by exhaustion, senescence, and decreased effector functions.^5^ The use of hiPSCs as a source for the generation of “off‐the‐shelf” CD8+ T cells holds the potential to enable large‐scale T cell‐based immunotherapy, overcoming limitations associated with culture time, T cell exhaustion, and decreased proliferative capacity.^5^, ^6^ However, the differentiation of lymphoid progenitors into mature naïve T cells and their selection are complex processes that heavily rely on the intricate architecture of the thymus with different cell types and the diverse composition of the extracellular matrix (ECM).^7^ Therefore, the in vitro generation of CD8+ T cells from hiPSCs is challenging and often involves co‐culture with murine OP9 cells bioengineered to express the delta like canonical NOTCH ligand 1 (DLL1) or DLL4.^8^, ^9^, ^10^, ^11^ Recent publications have also reported the formation of three‐dimensional aggregates of hiPSCs with murine stromal cells ectopically expressing DLL4, creating artificial thymic organoids (ATOs) for the induction of T cell differentiation.^12^, ^13^ There is an effort to replace OP9 cells due to the challenges associated with feeder‐based differentiation systems for clinical applications. Iriguchi et al.^14^ described a feeder‐free differentiation culture system based on the formation of embryoid bodies (EBs) in combination with immobilized DLL4 protein‐coated plates.

The efficiency of ACT relies on the specificity of tumor‐infiltrating lymphocytes (TILs) toward melanoma‐associated antigens. Antigens commonly overexpressed on melanoma cells include cancer germline antigens (CGAs) like NYESO‐1 or members of the MAGE‐A family of proteins, melanocyte differentiation antigens like melanoma‐associated antigen recognized by T cells (MART‐1), Melan‐A, glycoprotein 100 (gp100), tyrosinase, and melanoma‐associated chondroitin sulfate proteoglycan (MCSP) as well as neoantigens.^15^, ^16^, ^17^ Effective systems for the delivery of T cell receptors (TCRs) or chimeric antigen receptors (CARs) are non‐integrative DNA scaffold/matrix attachment region (S/MAR) vectors, which offer persistent expression of a gene of interest (GOI) over hundreds of cell divisions by binding to the chromosomal scaffold during the mitotic interphase.^18^, ^19^

In this study, we investigated two different strategies for differentiating hiPSCs transfected with S/MAR DNA vectors encoding a MART‐1 TCR or an MCSP CAR, respectively, into CD8+ single‐positive (SP) T cells via the hematopoietic stage. In the first approach, we utilized long‐term co‐culture with bioengineered OP9 stromal cells expressing important factors for T cell development to induce the differentiation of CD8+ T cells from CD34+ hematopoietic stem/progenitor cells (HSPCs). After 35 days of co‐culture, we successfully generated CD4 + CD8+ double‐positive (DP) T cells. However, the generation of CD8+ SP T cells was less efficient with this method. Substituting the OP9 cells with a T cell differentiation kit (Stemdiff, Stemcell Technologies) enabled the generation of CD8+ SP T cells after 47 days. Although the generated CD8+ SP T cells did not exhibit high expression of the T cell marker CD3, intracellular IFN‐γ and surface CD107a expression could be detected upon stimulation. Additionally, cytotoxic effects of the generated CD8+ SP T cells were observed after their co‐culture with melanoma cell lines expressing MART‐1 and MCSP.

## 2 METHODS

### 2.1 Cell culture

HiPSCs were reprogrammed from patient‐derived fibroblasts with the CytoTune™‐iPS 2.0 Sendai Reprogramming Kit (Thermo Fisher Scientific) and cultured in StemFit complete medium (Nippon genetics) supplemented with 100 ng/mL basic fibroblast growth factor (bFGF) (Peprotech) and 1:1000 normocin (InvivoGen) on matrigel‐(Corning) coated 6‐well plates. Before reaching 100% confluency, hiPSCs were passaged to fresh Matrigel‐coated plates using StemPro Accutase (GibcoLife Technologies) and kept in complete StemFit medium with 1:1000 rho‐associated protein kinase (ROCK) inhibitor for the first 24 h. Medium was changed every other/second day. For long time storage, hiPSCs were frozen in Bambanker freezing medium (Nippon genetics) and stored at −80°C or in liquid nitrogen.

The human melanoma cell lines WM266‐4 (RRID:CVCL_2765) and C32 [Human melanoma] (RRID:CVCL_1097) were purchased from ATCC and cultured in DMEM (Gibco®Life Technologies) containing 10% heat‐inactivated fetal bovine serum (FBS) (Biochrom), 1% penicillin/streptomycin (pen/strep) (Sigma‐Aldrich), 1% non‐essential amino acids (NEAA) (Sigma‐Aldrich), and 0.1 mM mercaptoethanol (Gibco®Life Technologies). Murine OP9 stromal cell lines (RRID:CVCL_4398) (provided by Dr. Pierre Guermonprez, Université de Paris) were maintained in Minimum Essential Medium (MEM) (Gibco®Life Technologies) containing 20% FCS, 1% pen/strep (Sigma‐Aldrich) and 0.1 mM mercaptoethanol (Gibco®Life Technologies) in gelatin‐(0.1%, Sigma‐Aldrich) coated flasks. Cultures were passaged just before reaching confluency. Co‐cultures of OP9 cells with hiPSCs were maintained in OP9 differentiation medium; in other words, MEM supplemented as above, except that 20% FCS was replaced with 10% FCS. All experiments were performed with mycoplasma‐free cells. All human cell lines have been authenticated using STR (or SNP) profiling within the last 3 years.

### 2.2 TCR/CAR vector preparation

S/MAR vector P17‐pSMARt‐Ele40‐CAG was kindly provided by Dr. Richard Harbottle (DNA vector Lab, DKFZ) and used as the backbone construct for further modifications with melanoma antigen‐specific receptor genes.^20^ The MART‐1‐TCR construct was kindly provided by Michael Nishimura.^21^ The MCSP‐CAR construct was kindly provided by Niels Schaft (Universitätsklinikum Erlangen).^22^ Both constructs were cloned into the S/MAR vector via the NheI and XhoI restriction sites.

### 2.3 Real‐time quantitave reverse transcription‐PCR

Total RNA isolation was performed using the RNeasy Mini Kit (Qiagen), according to the manufacturer's instructions. cDNA was generated from 500 ng total RNA by reverse transcription using the Revert Aid First Strand cDNA synthesis kit (Thermo Fisher Scientific), according to the manufacturer's instructions. Real‐time PCR was used to quantify gene expression with SYBR Green Master Mix (Applied Biosystems) and specific primers for the gene of interest. All PCR reactions were performed in triplicates and run on a 7500 Real‐Time PCR System device (Applied Biosystems). 18S expression was used as an endogenous control for the experiments. Results were analyzed using the ΔΔCt value method and the 7500 software, version 2.0.5 (Applied Biosystems).

### 2.4 Transfection and sorting of hiPSCs successfully transfected with S/MAR vectors

For transfection of the hiPSCs, Lipofectamine stem (Life technologies) was used according to the manufacturer's instructions. Briefly, hiPSCs were seeded in a Matrigel‐coated 24‐well plate in Stemfit complete medium + ROCK inhibitor 1 day before transfection. The transfection mix consisting of 25 μL Opti‐MEM + 1 μL Lipofectamine stem (Life technologies) mixed with 500 ng vector DNA diluted in 25 μL Opti‐MEM was added to cells after 10 min of incubation with ROCK inhibitor‐free medium. Medium changes were performed every other/second day, and cells were transferred to six‐well plates before reaching confluency. Using fluorescence microscopy, transfection efficiency was determined by quantifying mCherry expression.

### 2.5 Generation of T cells from hiPSCs using the OP9 co‐culture system

To induce the differentiation of hiPSCs, OP9 stromal cells were seeded onto 0.1% gelatine‐coated six‐well plates 1 day before use. The next day, hiPSCs were transferred to a confluent OP9 six‐well plate with OP9 differentiation medium. Half‐medium changes were performed by removing 1.5 mL of medium and adding 2 mL of fresh OP9 differentiation medium on days 4 and 7. On day 9, supernatant was collected and cells were incubated with 1 mL collagenase IV solution (1 mg/mL) and 1 mL 0.05% trypsin–EDTA for 20 and 10 min at 37°C, respectively, before collection. Cells and supernatant were centrifuged, resuspended in PBS and filtered through a 40 μm cell strainer. The obtained cell suspension was used for CD34+ HSPC isolation by fluorescence‐activated cell sorting or immunomagnetic cell separation using the EasySep human CD34 positive selection kit II (Stemcell Technologies) in combination with the EasySep magnet according to the manufacturer's instruction. Differentiation toward the CD4 + CD8+ DP stage was initiated by seeding CD34+ cells on confluent OP9‐FS12 monolayers in OP9 differentiation medium supplemented with 5 ng/mL IL‐7 (Peprotech) based on the protocol by Good et al.^23^ Medium changes were performed every second day. Non‐adherent cells were passaged every 5–7 days onto fresh confluent OP9‐FS12 monolayers. On day 44, non‐adherent cells were harvested and prepared for flow cytometric analysis of T cell marker expression (see Table 1).

**TABLE 1:** Antibody staining panel for the analysis of T cell markers on differentiating cells.

Laser	Filter	Specificity	Fluorochrome	Dilution	Company	Reference
355 nm	UV‐450/50	DAPI		1:5000	Roche	
405 nm	VL780/60	CD3	BV785	1:50	Biolegend	317,329
405 nm	VL710/50	CD8a	BV711	1:50	Biolegend	301,043
405 nm	VL450/50	TCRαβ	BV421	1:20	Biolegend	306,721
488 nm	BL525/50	mH‐2Kk	FITC (GFP)	1:100	Biolegend	116,304
561 nm	YG610/20	mCherry				
640 nm	RL780/60	CD4	APC‐Cy7	1:50	Biolegend	344,615
640 nm	RL670/30	CD45	APC	1:50	Biolegend	304,011

### 2.6 Generation of T cells using the STEMdiff T cell kit

For the generation of CD8+ SP T cells from hiPSCs, the STEMdiff T cell kit (Stemcell Technologies) was used according to the manufacturer's instructions. Briefly, 3.5 × 10^6^ hiPSCs were seeded into each well of a six‐well Aggrewell plate in EB formation medium and centrifuged. On days 2 and 3, half‐medium changes were performed. On day 5, EBs were harvested, collected on 40 μm cell strainers and transferred into non‐tissue culture‐treated six‐well with EB Medium B. Half‐medium changes were performed on days 7 and 10. On day 12, EBs were harvested and dissociated using collagenase II (2500 U/mL) and TrypLE Express (Thermo Fisher Scientific) for 20 min at 37°C. After centrifugation and resuspension in FACS Buffer, CD34+ HSPCs were isolated by Magnetic EasySep human CD34 positive selection according manufacturer's instructions. Isolated CD34+ HSPCs were analyzed by flow cytometry and further used for the differentiation into T cells. For that, 5 × 10^4^ cells were added per well in 50 μL Lymphoid Progenitor Expansion Medium into 24‐well plates that were pre‐coated with StemSpan Lymphoid Differentiation Coating Material for 2 h at RT. Half‐medium changes were performed on days 19 and 23. On day 26, cells were harvested and reseeded into freshly coated 24‐well plates at a density of 0.5–1 × 10^5^ cells/well in 500 μL T cell Progenitor maturation medium. Half‐medium changes were performed on days 30, 33, and 36 before the CD4 + CD8+ DP T cells were harvested on day 40 and further differentiated to CD8+ SP T cells. For that, 5 × 10^5^ CD4 + CD8+ DP T cells were seeded per well of a freshly coated 24‐well plate. CD8+ SP T cell maturation medium was supplemented with 10 ng/mL IL‐15 and 6.25 μL ImmunoCult CD3/CD28/CD2 T cell activator (Stemcell Technologies). Three days after seeding, 500 μL of fresh CD8+ SP T cell maturation medium (without activator) was added to the cells before the cells were harvested and analyzed by flow cytometry on day 7 after seeding (day 47 in total) or used for further functional experiments.

### 2.7 T cell stimulation

To evaluate degranulation and cytokine production of the differentiated T cells, they were stimulated with 20 ng/mL phorbol myristate acetate (PMA) and 1 μg/mL of ionomycin 4 h before harvest. After 1 h, 5 μg/mL brefeldin A was added to inhibit the protein transport. Unstimulated and stimulated cells were analyzed for surface expression of CD107a and intracellular expression of IFN‐γ by FACS using BD LSR Fortessa. Subsequent analysis of the acquired data was performed with FlowJo 7.2.2.

### 2.8 T cell cytotoxicity assay with human melanoma cell lines

CD8+ SP T cells were co‐cultured with melanoma cell lines C32 and WM266‐4 for 24–48 h to assess their cytotoxic capacity. Briefly, melanoma cells were labeled with 10 μM cell proliferation dye eFluor 450 (Invitrogen) for 20 min at RT in the dark before co‐culture. Afterwards, 5 × 10^4^ cells/well were seeded into a 24‐well plate and kept at 37°C for 24 h. The next day, CD8+ SP T cells were seeded on top of the melanoma cells at ratios of 1:5/10/20 (melanoma cells/T cells). After 24–48 h, cells were harvested using trypsin and stained with 2.5 μL propidium iodide (PI) (50 μg/mL) (BD Biosciences) per sample. Cells were analyzed by flow cytometry (BD LSR Fortessa) within 1 h after staining. Subsequent analysis of the acquired data was performed with FlowJo 7.2.2.

### 2.9 Statistics

Statistical evaluations were done using GraphPad Prism Version 5. Data are always presented as mean ± standard deviation (SD) and statistical significance is indicated using *p*‐values (*p < .05; **p < .01; ***p < .001). One‐way analysis of variance (ANOVA) was used to compare between multiple conditions and data sets. For the comparison of two groups, a two‐tailed student's *t* test was performed.

## 3 RESULTS

### 3.1 S/MAR vector‐transfected hiPSCs could be differentiated into CD34+ HSCPs after co‐culture with OP9 cells

**FIGURE 1:**
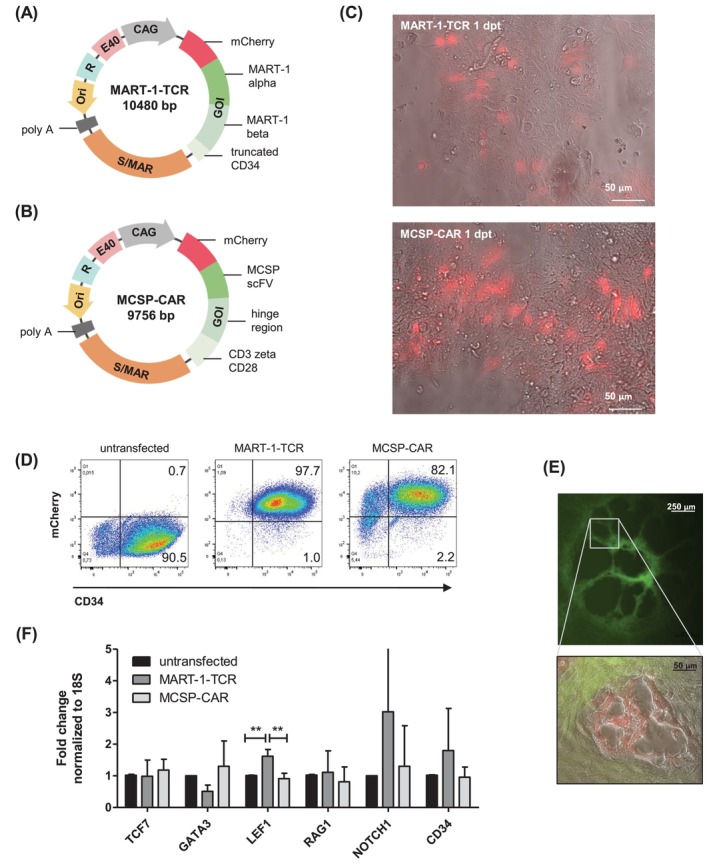
S/MAR DNA vectors coding for a MART‐1‐specific TCR and a MCSP‐specific CAR. (A,B) Vector map of the S/MAR vector containing the S/MAR element followed by a poly A tail. The GOI encodes a fusion protein consisting of mCherry as reporter gene and the alpha and beta chain of the TCR directed against MART‐1 (A) or the CAR with a scFv directed against MCSP, hinge region as well as CD3 zeta and truncated CD28 (B). (C) Fluorescent image of hiPSCs 1 dpt with a S/MAR vector coding for the MART‐1‐specific TCR or the MCSP‐specific CAR. The red fluorescence is due to the co‐expression of mCherry, which verifies the successful transfection. (D) Representative dot plots showing the percentage of CD34+ mCherry+ cells after immunomagnetic separation of untransfected cells and MART‐1‐TCR‐ or MCSP‐CAR‐transfected cells. (E) Representative microscopy image showing the development of cartwheel‐like mesoderm structures on day 9 of co‐culture of hiPSCs and OP9 stromal cells. (F) Fold changes of mRNA expression levels of T cell‐related differentiation genes in untransfected and MART‐1‐TCR‐ or MCSP‐CAR‐transfected hiPSCs on day 9 of differentiation (HSPCs). 18S was used as endogenous control. Data represent mean ± SD (*n* = 4).

To generate melanoma antigen‐specific T cells, S/MAR vectors were used in this study as the delivery system for the transfection of hiPSCs with a TCR or a CAR. Figure 1A illustrates the MART‐1‐TCR S/MAR vector encoding the α‐ and β‐chain of the melanoma antigen MART‐1 as the GOI, while Figure 1B shows the MCSP CAR S/MAR vector encoding the MCSP‐specific single chain variable fragment (scFv), the hinge region, an intracellular CD3 signaling domain together with a truncated CD28. Both vectors also include mCherry as a reporter gene for future selection processes. MCherry and the MART‐1‐TCR or the MCSP‐CAR, respectively, are linked via 2A peptides and are mutually expressed as polyproteins. The hiPSCs were transfected with the TCR or CAR S/MAR vector, respectively, and the mCherry expression was analyzed 1 day post transfection (dpt) by fluorescence microscopy to determine transfection efficiency (Figure 1C). FACS 6 dpt revealed that 8% and 33% of live cells were mCherry+ positive upon transfection. Additional rounds of sorting were conducted to establish a cell line with a stable TCR or CAR expression (Figure S1A).

The generation of CD34+ HSPCs was facilitated by co‐culturing hiPSCs with confluent GFP+ murine bone marrow‐derived OP9 stromal cells for 9 days. CD34+ HSPCs were isolated from the emerging cartwheel‐like mesodermal structures using immunomagnetic cell separation (Human CD34 Positive Selection kit) (Figure 1E). Figure 1D shows a representative example of the analysis of isolated CD34+ cells among live cells. On average, the frequency of CD34+ mCherry+ cells varied between 98% for MART‐1‐transfected cells and 82% for MCSP‐transfected cells. Untransfected CD34+ cells did not exhibit mCherry expression.

To assess any potential effects of TCR/CAR transfection on the expression of T cell‐related differentiation genes, RT‐qPCR analysis was performed (Figure 1F). The gene expression levels of TCF7, encoding the transcription factor T cell factor‐1 (TCF1), and recombination activation gene 1 (RAG1), crucial for generating the antigen receptor repertoire, did not show significant changes between untransfected, MART‐1‐TCR‐transfected, and MCSP‐CAR‐transfected CD34+ cells.^24^, ^25^ The expression level of GATA‐binding protein 3 (GATA3), which induces T cell lineage commitment of thymic progenitors together with NOTCH1, was slightly decreased in MART‐1‐TCR‐expressing CD34+ cells compared to untransfected and MCSP‐CAR‐transfected cells.^26^ However, this difference was not significant. In contrast, a significantly higher expression of lymphoid enhancer‐binding factor 1 (LEF1), which promotes β‐selection together with TCF1, was detected in MART‐1‐TCR‐expressing cells.^27^ A similar trend, albeit not significant, was observed for the NOTCH1 expression, which is important for the development of CD4 + CD8+ DP T cells in the thymus.^24^, ^28^ Based on these results, we assume that there is no suppression of T cell‐related differentiation in TCR/CAR‐transfected cells during the differentiation process.

### 3.2 Co‐culture of CD34+ HSPCs on bioengineered OP9 cells induced CD4 + CD8+ DP T cell lineage commitment

**FIGURE 2:**
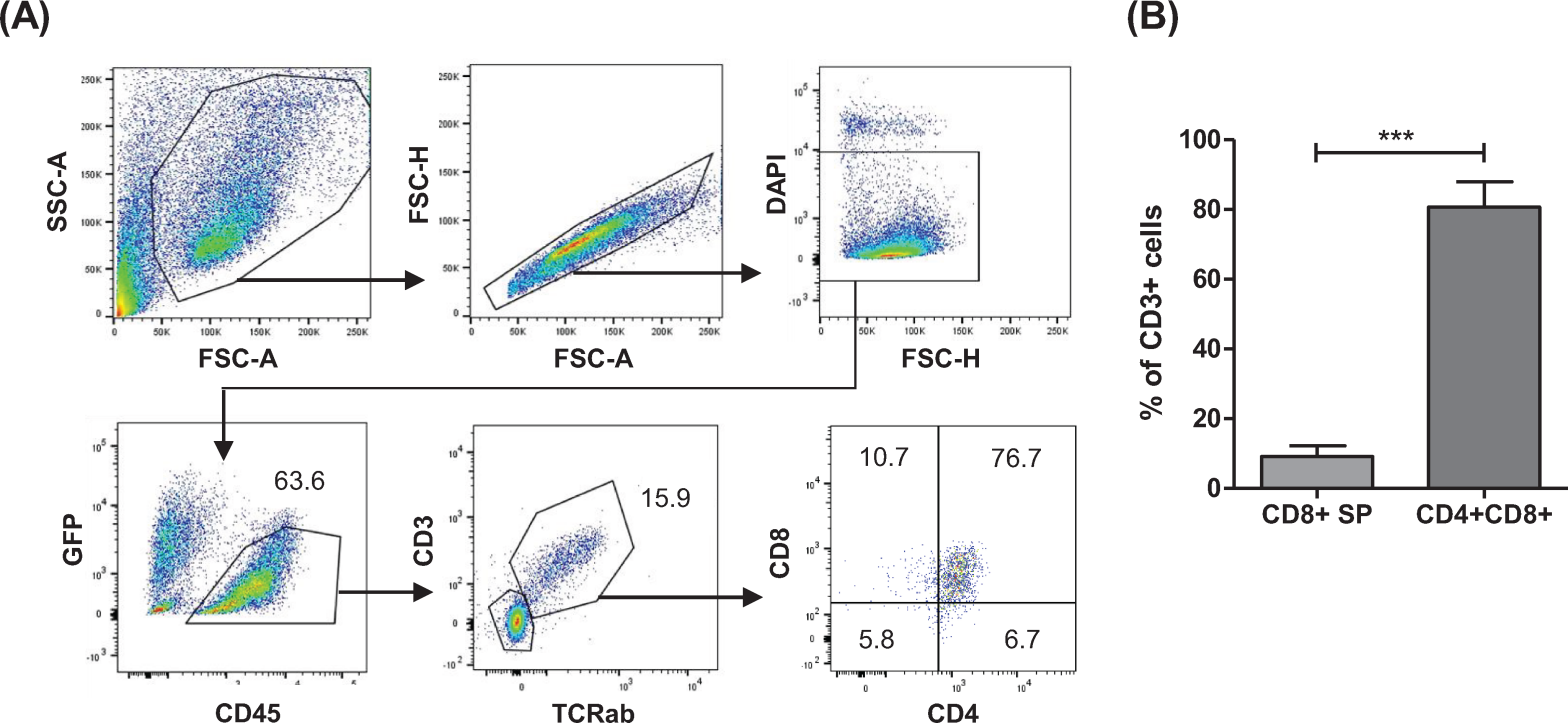
The OP9 co‐culture system enables the differentiation of CD4+ CD8+ DP T cells from CD34+ HSPCs. (A) Representative flow cytometry profile of differentiating, untransfected cells on day 44 of co‐culture. Cells were gated on live GFP‐CD45+ cells expressing CD3, TCRαβ, CD4, and CD8. Cells were cultured on OP9‐FS12hDLL4 in medium supplemented with recombinant IL‐7. (B) Bar diagram showing the percentage of CD8+ SP and CD4 + CD8 DP T cells from four independent experiments gated on CD3+ cells. Data represent mean ± SD (*n* = 4). ***p < .005.

To further induce T cell differentiation, isolated CD34+ cells from day 9 of co‐culture were seeded on top of confluent OP9‐FS12hDLL4 cells bioengineered to express the FLT3‐L, SCF, CXCL12, and hDLL4 in medium supplemented with IL‐7. Semiadherent cells were analyzed for T cell marker expression by FACS after 35 days in co‐culture, as shown in Figure 2A. T cell lineage commitment of CD34+ HSPCs was indicated by the presence of CD45 + CD3 + TCRab+ cells (Figure 2A). On average, the frequency of developing CD4 + CD8+ DP T cells among CD3 + TCRab+ cells ranged from 72% to 89%. Therefore, the in vitro co‐culture of CD34+ HSPCs on OP9‐FS12hDLL4 cells in medium supplemented with recombinant IL‐7 facilitated the differentiation of CD4 + CD8+ DP T cells. However, the maturation of CD8+ SP T cells remained insufficient using this OP9 co‐culture system since less than 10% of the CD3+ cells were identified as CD8+ SP (Figure 2B).

### 3.3 The feeder‐free differentiation kit induced CD8+ SP T cell lineage commitment

**FIGURE 3:**
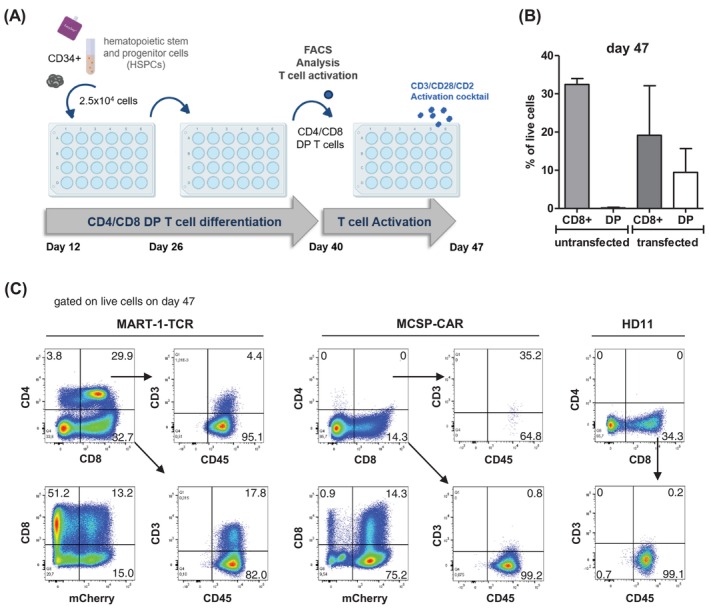
Generation of CD8+ SP T cells from CD34+ HSPCs. (A) Schematic representation of the differentiation process of hiPSCs into CD4+ CD8+ DP T cells and the subsequent activation of these T cells. (B) Percentage of CD8+ SP and CD4+ CD8+ DP T cells within the live cell population on day 47 of the differentiation process of untransfected (*n* = 2) and transfected (*n* = 4) (MART‐1‐TCR and MCSP‐CAR) hiPSCs to T cells analyzed by flow cytometry. (C) Representative dot plots of differentiated cells on day 47 showing the gating strategy for the identification of live cells that express CD4, CD8 as well as mCherry as a marker for the MCSP‐CAR and the MART‐1‐TCR, respectively. CD8+ population was further analyzed for CD45 and CD3 expression. MCherry expression was investigated in the total live population.

Feeder cell‐based systems for generating T cells are less suitable for clinical applications. Therefore, alternative feeder‐free methods provide better prospects for future cancer treatment strategies. In this study, the STEMdiff T Cell Kit (Stemcell Technologies) with serum‐free media and supplements was used to generate CD34+ HSPCs from hiPSCs through the formation of EBs followed by 28 days of culture on lymphoid coating material in lymphoid progenitor medium. To induce the differentiation of CD8+ SP T cells, the cells were treated with an activation cocktail comprising CD3, CD28, and CD2 in combination with IL‐15 for 7 days (Figure 3A). The 47‐day differentiation process resulted in the development of CD8+ SP T cells from MART‐1‐TCR and MCSP‐CAR‐transfected as well as untransfected hiPSCs. Among the total live cell population, 32.7% of MART‐1‐TCR‐transfected cells and 34.3% of untransfected cells expressed CD8 but not CD4. One representative example for MCSP‐transfected cells showed the detection of 14.3% CD8+ SP T cells (Figure 3C). On average, for untransfected live cells about 32% expressed CD8 but not CD4 on day 47 of differentiation. For transfected cells (MART‐1‐TCR and MCSP‐CAR) about 20% CD8+ SP cells were detected (Figure 3B). The analysis of the expression of the T cell surface marker CD3 revealed that only 17.8% of CD8+ SP MART‐1‐TCR‐transfected cells and 0.8% of CD8+ SP MCSP‐CAR‐transfected cells co‐expressed CD3. In contrast, almost 100% of the CD8+ SP T cells were positive for CD45. MCherry expression, which is equivalent to MART‐1‐TCR or MCSP‐CAR expression, respectively, was detected in 28.2% (MART‐1‐TCR) and 89.5% (MCSP‐CAR) of the live population, respectively (Figure 3C).

### 3.4 Differentiated CD8+ SP T cells showed IFN‐γ and CD107a expression after stimulation and exhibited cytotoxic effects against melanoma cells

**FIGURE 4:**
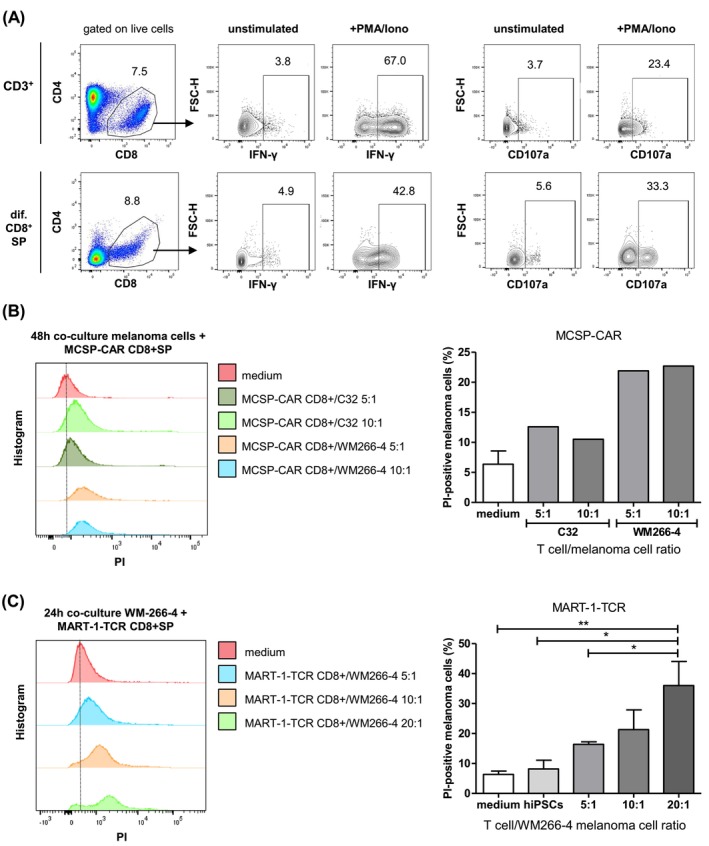
Examination of the effector function of hiPSC‐derived CD8+ SP T cells. (A) Flow cytometry analysis of degranulation (CD107a) and intracellular IFN‐γ expression of CD8^+^ SP T cells after 47 days of differentiation. Cells were additionally stimulated with PMA/iono 4 h prior to harvest and treated with brefeldin A after 1 h. Numbers show the percentage of IFN‐γ‐ or CD107a‐positive cells. (B) Quantification of the amount of apoptotic C32 and WM266‐4 melanoma cells by PI staining after co‐culture with hiPSCs (control) or MCSP‐CAR‐transfected CD8+ SP cells for 48 h with different T cell/melanoma cell ratios. Bar diagram displaying the quantities of PI‐positive melanoma cells (C32 or WM266‐4) after co‐culture. Melanoma cells were labeled with CP‐Dye450 (Biosciences) beforehand for distinction. Data represent mean ± SD (*n* = 4, medium group; *n* = 1 all other groups). (C) Quantification of the amount of apoptotic WM266‐4 melanoma cells by PI staining after co‐culture with hiPSCs (control) or MART‐1‐TCR‐transfected CD8+ SP cells for 24 h with different melanoma cell/T cell ratios. Bar diagram displaying the quantities of PI‐positive, dead WM266‐4 melanoma cells after co‐culture. Melanoma cells were labeled with CP‐Dye450 (Biosciences) beforehand for distinction. Data represent mean ± SD (*n* = 2, hiPSC group; *n* = 4, all other groups). *p < .05, **p < .01.

The secretion of cytokines like IFN‐γ, granzymes, and perforin represents a potent effector function of CD8+ T cells in anti‐tumor immunity.^29^ In this study, we examined the surface expression of CD107a and intracellular IFN‐γ expression in generated CD8+ SP T cells upon stimulation with PMA and ionomycin (iono) using flow cytometry. As shown in Figure 4A, the stimulation with PMA/iono induced IFN‐γ expression in CD3+ T cells isolated from human PBMCs as well as in differentiated CD8+ SP T cells. Similar results were observed for the expression of the degranulation marker CD107a. Following PMA/iono stimulation, the frequency of CD107a‐expressing differentiated CD8+ SP T cells increased from 5.6% to 33.3% in the unstimulated control. The cytotoxic function of the generated CD8+ SP T cells was evaluated in cytotoxicity assays with C32 and WM266‐4 melanoma cells expressing MART‐1 and MCSP. As shown in Figure 4B, the frequency of PI‐positive cells increased for both C32 and WM266‐4 upon addition of MCSP‐CAR‐transfected CD8+ SP T cells compared to medium control. For WM266‐4, a lower melanoma cell/T cell ratio (1:10) resulted in a slightly higher cytotoxicity compared to a higher ratio (1:5). In the case of C32 cells, no significant difference in the frequency of dead cells was observed. In contrast, the 1:5 ratio of melanoma cells to MCSP‐CAR‐transfected CD8+ T cells stimulated higher cytotoxicity of WM266‐4 cells than C32 cells. This indicates that the MCSP‐CAR‐transfected CD8+ T cells exerted a stronger cytotoxic effect against WM266‐4 cells compared to C32 cells. Further FACS analysis revealed a higher percentage of WM266‐4 cells expressing MCSP on their surface compared to C32 cells (Figure S1B).

WM266‐4 cells were selected for the cytotoxicity assay with differentiated MART‐1‐TCR CD8+ SP T cells (Figure 4C). The frequency of dead cells increased depending on the ratio between tumor target cells and MART‐1‐TCR‐transfected CD8+ SP T cells co‐cultured for 24 h compared to co‐culture with undifferentiated hiPSCs or a medium control. The highest and statistically significant cytotoxic effect was observed at a 1:20 melanoma cell/T cell ratio, resulting in 36% dead WM266‐4 cells. For a 1:5 and 1:10 melanoma cell/T cell ratio, the effect was still not significant, with about 15% or 22% dead cells, respectively. In conclusion, hiPSC‐derived CD8+ SP T cells demonstrated cytotoxic effects against melanoma cells after co‐culture for 24 or 48 h. However, a higher ratio of T cells to melanoma cells was necessary to achieve a statistically significant cytotoxicity.

## 4 DISCUSSION

The development of effective treatment strategies for cancer patients remains a topic of great interest.^30^ In this study, we utilized hiPSCs transfected with S/MAR vectors as a source for the generation of melanoma antigen‐specific CD8+ SP T cells. We selected S/MAR DNA vectors as our gene delivery system due to their resistance to epigenetic silencing during differentiation processes, their non‐integration into the host genome, and their ability to sustain a constitutive stable expression of our GOI.^31^, ^32^, ^33^ Using S/MAR vectors, we successfully generated hiPSCs that stably expressed a MART‐1‐specific TCR or a MCSP‐specific CAR, respectively. However, multiple rounds of sorting were necessary to enrich these cells. It might be because the vector needs to find the right nuclear location close to a transcription factory to achieve stable expression.^31^, ^32^, ^33^ As expected, the transfection with the vector had minimal impact on the expression of T cell‐related differentiation genes at the CD34+ HSPC stage. The most significant differences in expression levels between transfected and untransfected cells were observed for LEF1 and NOTCH1. However, it is important to note that LEF1 is only minimally expressed at the CD34+ stage, and it would be beneficial to include additional developmental stages, such as CD4 + CD8+ DP T cells, in the gene expression analysis.

In general, we demonstrated that differentiated hiPSCs maintained the expression of the TCR or CAR not only at the CD34+ stage but also at the CD8+ SP level. The use of OP9 cells is a widely employed method to induce hematopoietic specification of hiPSCs or ESCs in vitro.^34^, ^35^, ^36^ Further differentiation of the CD34+ HSPCs can be achieved by adding important factors involved in T cell differentiation such as IL‐7, FLT3L, SCF, and chemokines like CXCL12 and CCL25.^37^ While we successfully generated CD4 + CD8+ DP T cells from CD34+ HSPCs using OP9‐FS12hDLL4 cells, we encountered challenges in generating CD8+ SP T cells. This may be attributed to the absence of selection steps that normally occur in specialized niches within the human thymus involving antigen‐presenting cells and other factors.^38^ Additionally, the OP9 approach lacks the intricate 3D organization present in the human thymus. Hence, novel organoid‐based T cell differentiation methods, such as ATOs, offer promising opportunities for studying T cell development in a 3D environment. However, even cells generated through these approaches exhibit partial deficiencies in in vivo immunocompetence upon engraftment.^12^, ^13^, ^39^ Other studies have focused on the coordinated expression of specific transcription factors to induce T lymphopoiesis or culture on plates coated with immobilized DLL4 protein.^14^, ^39^

In our study, we successfully employed a T cell differentiation kit to generate CD8+ SP T cells, albeit in lower quantities. Unexpectedly, despite expressing CD8 on day 47 of differentiation, the cells were partly lacking the expression of the T cell marker CD3. Similar observations have recently been described by Van der Stegen et al.^40^ during the generation of T cells from iPSCs. It was demonstrated that premature CAR expression at the early double‐negative (DN) stage of T cell development interfered with NOTCH signaling and, consequently, hindered the induction of the transition from DN to DP via DLL4.^40^, ^41^, ^42^

Although the generated CD8+ SP T cells lacked CD3 expression, they exhibited intracellular expression of IFN‐γ and upregulated CD107a expression upon PMA/iono stimulation. Instead of activating the TCR/CD3 complex, PMA/iono stimulation induces several intracellular signaling pathways for cytokine production via activation of protein kinase C and due to iono's function as a calcium ionophore.^43^ To specifically investigate TCR‐dependent activation, the use of MART‐1 tetramers could be employed. One limitation for the allogeneic use of “off‐the‐shelf” T cells is the requirement for HLA matching. HLA class I molecules, including HLA‐A, ‐B, and ‐C, are expressed on all nucleated cells and recognized by CD8+ T cells.^44^ For the cytotoxicity study, we used HLA‐A*02:01 WM266‐4 melanoma cells since the MART‐1 receptor was HLA‐A*02 restricted (Figure S1C). We demonstrated a significant increase in the frequency of dead WM266‐4 cells after 24 h of co‐culture with MART‐1‐TCR‐transfected CD8+ SP T cells at a ratio of 1:20. To achieve an even stronger cytotoxic effect, increasing the ratio of T cells to melanoma cells could be considered. Another possibility would be to sort the cells harvested on day 47 of differentiation for the presence of CD8 and thereby removing undifferentiated and dead cells. The stronger cytotoxic effect of MCSP‐CAR‐transfected CD8+ SP T cells toward WM266‐4 cells compared to C32 cells could be attributed to the higher frequency of WM266‐4 cells expressing MCSP on their surface in comparison with C32 cells. To overcome HLA restrictions in clinical approaches, potential strategies include the use of genome‐editing technologies to generate immunocompatible donor hiPSCs, such as by knocking out the β2‐microglobulin (B2M) gene, which is essential for HLA class I expression.^44^, ^45^, ^46^, ^47^

In conclusion, the in vitro generation of antigen‐specific CD8+ SP T cells holds promise for the improvement of ACT. However, there are still several unresolved questions that need to be addressed before hiPSC‐derived antigen‐specific T cells can be effectively utilized for the treatment of melanoma patients.

## AUTHOR CONTRIBUTIONS

**Juliane Poelchen:** Conceptualization; formal analysis; investigation; methodology; project administration; visualization; writing – original draft. **Sandra Pardo:** Writing – review and editing. **Daniel Novak:** Writing – review and editing. **Qian Sun:** Writing – review and editing. **Tamara Steinfass:** Writing – review and editing. **Marlene Vierthaler:** Writing – review and editing. **Özge Cicek Sener:** Writing – review and editing. **Karol Granados Blanco:** Writing – review and editing. **Yiman Wang:** Writing – review and editing. **Jan Peter Nicolay:** Writing – review and editing. **Pierre Guermonprez:** Writing – review and editing. **Richard Harbottle:** Writing – review and editing. **Viktor Umansky:** Supervision; writing – review and editing. **Jochen Utikal:** Conceptualization; funding acquisition; supervision; writing – review and editing.

## FUNDING INFORMATION

This project was funded by the Deutsche Forschungsgemeinschaft (DFG, German Research Foundation)—project numbers 259332240/RTG 2099 and 676288/UT 112/1–1.

## CONFLICT OF INTEREST STATEMENT

The authors declare that there are no conflicts of interest.

## ETHICS STATEMENT

This study involves hiPSC lines reprogrammed from patient‐derived fibroblasts and was approved by the ethics committee II of the University of Heidelberg (2009‐350N‐MA). Donors of these fibroblasts gave informed consent to participate in the study before taking part.

## Supporting information


**Figure S1.** A percentage of mCherry+ hiPSCs at different time points after.

## Data Availability

The data that support the findings of this study are available from the corresponding author upon reasonable request.
