# Supplementary material for: Generation and functional analysis of melanoma antigen‐specific CD8+ T cells derived from S/MAR vector‐transfected human induced pluripotent stem cells
Source: Int J Cancer. 2025 Jun 12;157(9):1876–87. doi: 10.1002/ijc.35524 (PMC12407039; doi:10.1002/ijc.35524)
Supplement: Supplementary file 1 — Figure S1. A percentage of mCherry+ hiPSCs at different time points after. [file IJC-157-1876-s001.pdf]

Generation and functional analysis of melanoma antigen-specific CD8+ T cells derived from S/MAR vector-transfected human induced pluripotent stem cells

Juliane Poelchen, Sandra Pardo, Daniel Novak, Qian Sun, Tamara Steinfass, Marlene Vierthaler, Özge Cicek Sener, Karol Granados Blanco, Yiman Wang, Jan Peter Nicolay, Pierre Guermonprez, Richard Harbottle, Viktor Umansky, Jochen Utikal

Table of content

Supplementary Figure 1

(A)

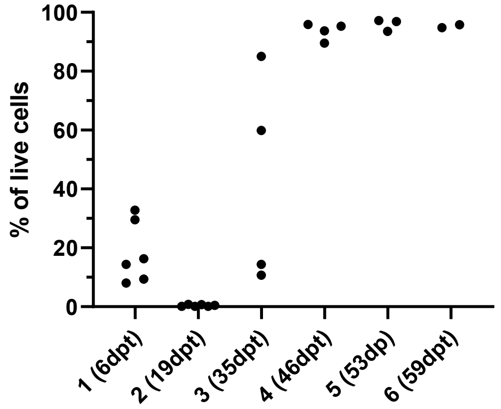

(B)

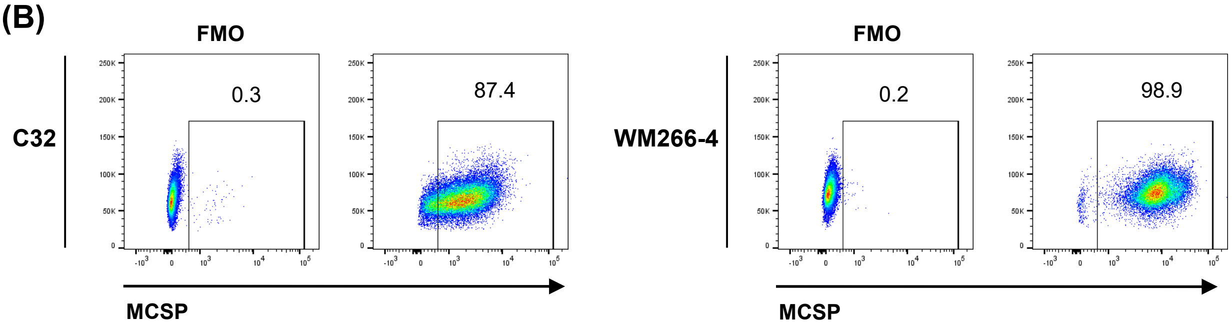

(C)

Cell line	HLA-A (allele 1)	HLA-A (allele 2)
hiPSC (HD11)	**11**:01:01G	**68**:01:02G
C32	**01**:01:01G	**24**:02:01G
WM266-4	**02**:01:01G	**29**:02:01G
MART-1 TCR	HLA-A*02	

Supplementary Fig. 1.: A Percentage of mCherry+ hiPSCs at different time points after transfection with either the MART-1-TCR or the MCSP-CAR construct quantified by FACS. **B** Representative flow cytometry dot plots of C32 and WM266-4 melanoma cells after MCSP surface staining. Fluorescence minus one (FMO) control was used to set the positive gates. Numbers indicate the percentage of MCSP-positive cells from live cells. **C** Sequencing results for the analysis of the HLA-A alleles.
